# Posterolateral Knee Ligament Reconstruction Using the Arciero Technique Provides Greater Rotational Stability Than the Modified Larson Technique: A Biomechanical Study

**DOI:** 10.1177/03635465241294072

**Published:** 2025-01-01

**Authors:** Christian Coppola, Maximilian Sigloch, Romed Hoermann, Michael Schlumberger, Philipp Schuster, Werner Schmoelz, Raul Mayr

**Affiliations:** * Department of Orthopedics and Traumatology, Medical University of Innsbruck, Innsbruck, Austria; † Institute for Clinical Anatomy, Medical University of Innsbruck, Innsbruck, Austria; ‡ Centre for Sports Orthopedics and Special Joint Surgery, Orthopaedic Hospital Markgroeningen, Markgroeningen, Germany; § Department of Orthopedics and Traumatology, Paracelsus Medical University, Clinic Nuremberg, Nuremberg, Germany; Investigation performed at the Biomechanics Laboratory of the Department of Orthopadics and Traumatology, Medical University of Innsbruck, Innsbruck, Austria

**Keywords:** posterolateral corner reconstruction, Larson, Arciero, biomechanics

## Abstract

**Background:**

It is still unknown if the double–femoral tunnel technique (Arciero [ARC]) provides better stability as compared with the single–femoral tunnel technique (modified Larson [LAR]) in posterolateral corner reconstruction. The ideal angle of fixation of the popliteofibular strand in ARC is also unknown.

**Hypotheses:**

The ARC provides greater external rotation (ER) stability than the LAR (hypothesis 1); there is no difference in varus rotation (VR) stability between LAR and ARC (hypothesis 2); and femoral fixation of the popliteofibular strand at 60° during the ARC leads to greater ER stability than fixation at 30° or 90° of knee flexion (hypothesis 3).

**Study Design:**

Controlled laboratory study.

**Methods:**

Eight fresh-frozen human knees were tested in a knee test bench in 4 states: native, posterolateral deficiency, LAR, and ARC. With the ARC, the popliteofibular strand was fixed at 30°, 60°, and 90° (ARC30, ARC60, ARC90). The order of testing (LAR/ARC) was randomized. A tibial ER and VR torque of 5 N·m was applied at 0°, 30°, 60°, and 90°. Rotation degrees were captured using an ultrasound-based analysis system. Wilcoxon signed rank tests were used to assess statistical significance between paired groups in different states.

**Results:**

The ARC and LAR significantly improved VR and ER stability at all flexion angles in comparison with posterolateral deficiency (*P* < .05). At 60° and 90°, ARC30 showed significantly greater ER stability in comparison with the LAR (mean ± SD; ARC30 vs LAR at 60°, 21.2°± 5.1° vs 15.4°± 5.6° [*P* < .05]; ARC30 vs LAR at 90°, 23.7°± 5.6° vs 16.8°± 6.3° [*P* < .05]). At 90°, the LAR showed significantly greater VR instability in comparison with the native state (3.5°± 1.5° vs 2.5°± 1.0°; *P* = .012), and ARC30 was not significantly different from the native state with respect to VR (2.9°± 1.5° vs 2.5°± 1.0°; *P* = .327). No significant differences in ER and VR were found among ARC30, ARC60, and ARC90 at any flexion angle (*P*≥ .05).

**Conclusion:**

The ARC technique provided greater tibial ER stability in comparison with the LAR at higher flexion angles (hypothesis 1 accepted). There were no differences between LAR and ARC in restoring VR stability, except at 90° (hypothesis 2 partly accepted). Different femoral flexion angles for fixation of the popliteofibular strand during the ARC did not show any significant differences in relation to knee stability (hypothesis 3 rejected).

**Clinical Relevance:**

Posterolateral corner reconstruction using the ARC technique provides greater ER stability at higher flexion angles than the modified LAR technique.

Injuries to the posterolateral corner (PLC) of the knee represent severe trauma and are often associated with cruciate ligament tears.^7,8,11^ The primary structures in the PLC are the lateral collateral ligament (LCL), popliteus tendon (PT), and popliteofibular ligament (PFL).^1^ These act as primary restraints against tibial external rotation (ER), varus rotation (VR), and, to a lesser extent, posterior translation of the tibia.^12,21,28^ Surgical treatment of PLC injuries continues to be challenging and is associated with a high risk of residual knee instability.^16,19,27,29^ Various PLC reconstruction techniques have been described using tendon grafts.^15,20,27,30^ Fibular tunnel–based PLC reconstruction techniques are widely used and can be performed with single or double femoral tunnels for graft fixation.^4,10^

In the modified Larson technique (LAR), both ends of the tendon graft, which passes through a fibular tunnel, are fixed at the most isometric point on the lateral condyle of the femur.^18^ In 2005, Robert Arciero described a double–femoral tunnel technique (ARC) that aimed to restore the native femoral attachments of the PLC.^4^ The graft strands are fixed at the native insertion sites of the LCL and PT. In comparison with the LAR technique, this technique can be regarded as a more anatomic approach to improve tibial ER stability. In the original description of the ARC technique, the method provides final fixation of both femoral strands—the LCL strand and the popliteofibular strand (PFS)—at approximately 30° of knee flexion.^4^ However, experimental studies have shown that the highest mean load response for ER for the PT and the PFL is at 60° of knee flexion.^17^ The ideal tensioning flexion angle for the PFS in the ARC technique is therefore still unclear and has not yet been investigated.

The aim of the present study was to carry out a biomechanical comparison of the LAR and ARC techniques with regard to ER and VR stability. In addition, fixation of the PFS in the ARC technique at 30°, 60°, and 90° of knee flexion was compared. The first hypothesis was that PLC reconstruction using the ARC would lead to better ER stability in comparison with the LAR. The second hypothesis was that there is no difference between the LAR and ARC techniques in relation to VR stability at various degrees of flexion. The third hypothesis was that femoral fixation of the PFS at 60° during the ARC would lead to greater ER stability than fixation at 30° or 90° of knee flexion.

## Methods

### Graft and Specimen Preparation

Eight fresh-frozen human knee joints from 8 donors with a median age of 86 years (range, 70-92 years; 4 men, 4 women) were used for this biomechanical study. The bodies were donated to the local anatomic institute by individuals who had provided informed consent before death to the use of their bodies for scientific and educational purposes.^6^ For each knee, the LAR and ARC were performed in a randomized fashion with a separate tendon graft. Computed tomography was performed to exclude severe knee arthritis (Kellgren-Lawrence ≥3). A medial arthrotomy was performed to check the integrity of both cruciate ligaments. The joint was closed with a suture. Before testing, the knees were stored at −20°C and thawed at room temperature the day before testing to allow skin and subcutaneous tissue resection.

Bovine tendons (extensor digitorum communis) were used as grafts to allow uniform size preparation.^9,31^ A graft 22 cm long and 4.5 mm in diameter was prepared. Both free ends were armed using a Krakow suture type with a nonabsorbable high-strength suture (FiberWire No. 2; Arthrex Inc). The grafts were preconditioned with a load of 50 N for 5 minutes on the graft preparation table (Graft Prep Station; Arthrex Inc). A different tendon was used for each of the 2 reconstruction techniques.

After ensuring that the lateral/medial ligamentous apparatus and the extensor apparatus and cruciate ligaments were intact, the knee flexion axis was determined using the Stannard and Schmidt method.^24^ The femur and the tibia were osteotomized at 150 mm from the flexion axis. The fibula was secured against rotation on the tibia using 2 screws.^2^ The femur and tibia were embedded in a standardized manner using polymethylmethacrylate (PMMA) cement (Technovit 3040; Kulzer GmbH). Embedding was performed using a custom-made rig that allows natural alignment of each knee. After curing of the embedding polymer, the knee was positioned in a custom-made test bench.^14^ After final alignment of the joint in the test bench, each knee was preconditioned by flexing and extending it 10 times.

### Motion Analysis

**Figure 1.**
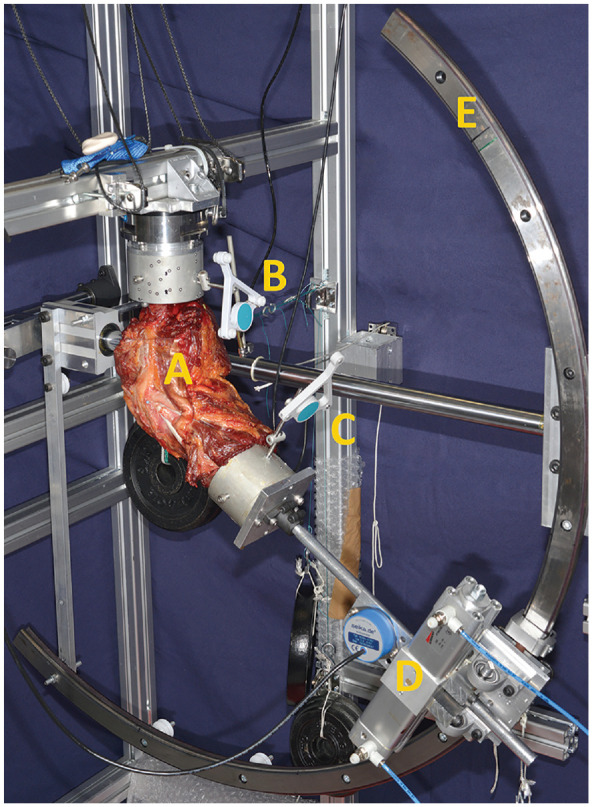
A specimen loaded in a custom-made test bench that allows motion with 6 degrees of freedom. (A) Specimen. (B) Femoral motion measurement sensor. (C) Tibial motion measurement sensor. (D) Pneumatic rotatory cylinder. (E) Test bench guidance rail.

A custom-made test bench^14^ that allows motion with 6 degrees of freedom was used (Figure 1). Motion of the tibia at 0°, 30°, 60°, and 90° of flexion relative to the fixed femur was recorded. ER torque was applied by a pneumatic rotatory cylinder (Camozzi Automation GmbH), and VR torque was induced using a pulley-and-weight system. The ER and VR of the tibia relative to the femur were measured using an ultrasound-based 3-dimensional motion analysis system with a rotational measurement accuracy of 0.1° (Winbiomechanics; Zebris).^22^ Measurement sensors were affixed to the posterior side of the femur and tibia. The specimens were kept moist throughout the duration of testing. Kinematic data were processed using the Winbiomechanics software program (Zebris).

### Testing Protocol

The testing protocol comprised the following states: native state (NAT), posterolateral deficiency, LAR, ARC with PFS fixation at 30° of flexion (ARC30), ARC with PFS fixation at 60° of flexion (ARC60), and ARC with PFS fixation at 90° of flexion (ARC90). Static investigation of knee joint stability was carried out at 0°, 30°, 60°, and 90° of flexion by applying 5 N·m of tibial ER torque and 5 N·m of VR moment.^3,19^ The sequence for the reconstructions was randomized between the third and fourth states. After testing of the first PLC reconstruction technique, the femoral bone tunnels were filled with PMMA cement to allow for new femoral bone tunnel drilling.^19^

### Establishing Posterolateral Deficiency

To simulate grade III posterolateral instability, the PT and LCL were cut at the femoral attachment and the PFL at the fibular attachment, using a scalpel.^20^

### Fibular Tunnel Creation

Immediately below the head of the fibula, the ventral soft tissue was detached with the raspatory at the transition from the shaft of the fibula head. The dorsal portions of the fibular head were also carefully exposed. A K-wire was drilled from anterolateral in a posteromedial direction. After checking of the correct position of the K-wire using a fluoroscope, a 5-mm tunnel was drilled.

### PLC Reconstruction Using the Modified LAR Technique

**Figure 2.**
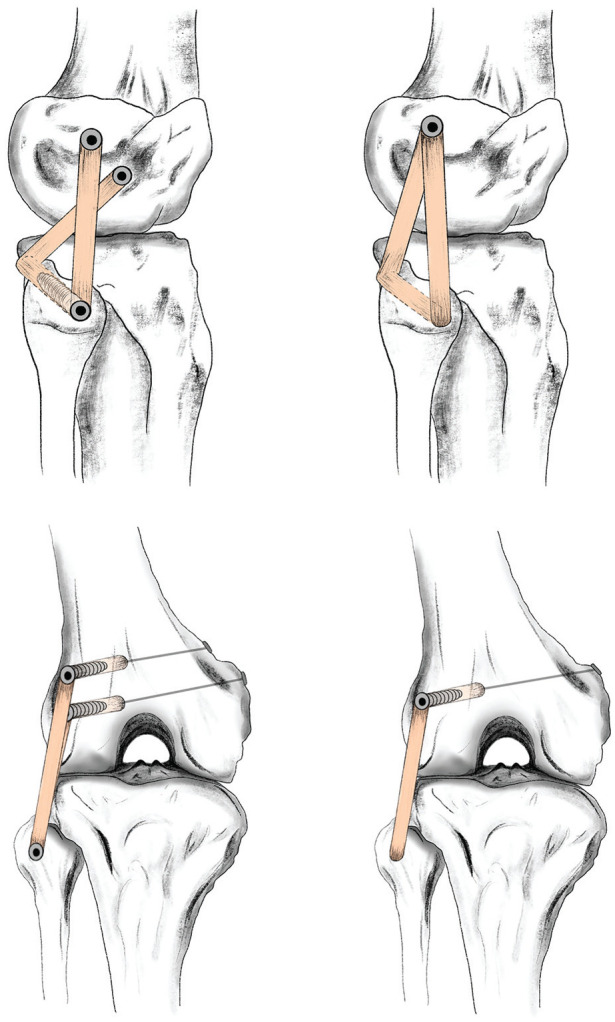
Schematic depiction of reconstruction of the posterolateral complex of the knee. The top row shows a lateral view of the Arciero technique and the modified Larson technique (from left to right). The bottom row shows an anterior-posterior view of both techniques in analogous order.

An incision was made over the lateral epicondyle, and the iliotibial tract was divided along its fibers. A K-wire was drilled slightly anterior to the lateral epicondyle in a slightly proximal, anterior direction.^18^ Isometry of the femoral insertion point was then verified while the knee joint was moved through a range of motion from 0° to 120° of flexion. Once the most isometric point was detected, an 8-mm socket tunnel was created to the opposite cortical bone. The graft was first pulled through the fibular canal, and both ends were brought to the same length. The dorsal graft limb was passed under the biceps femoris muscle and brought out to the femoral side. The 2 graft limbs were adjusted to the same length and pulled medially through the femoral socket passage. The 2 limbs were tensioned separately. A tensile force of 25 N was applied to each of the 2 graft limbs. Femoral fixation was achieved by inserting a 9 × 30–mm PEEK interference screw (Arthrex Inc) at 60° of flexion, without any external forces acting on the tibia.^18^ After previous aperture graft fixation, additional medial cortical fixation was carried out using a cortical button (Endobutton; Smith and Nephew plc) (Figure 2) to guarantee position of the graft while testing.

### PLC Reconstruction Using the ARC Technique

An incision was made over the lateral epicondyle, and the iliotibial tract was divided along its fibers. PLC reconstruction with 2 femoral tunnels was performed using the technique described by Arciero.^4^ To create the LCL attachment, a guide wire was passed approximately 3 to 4 mm anterior to the central femoral insertion of the LCL. A 6-mm socket tunnel was drilled toward the medial cortex. The PFS tunnel was created in the same way, 3 to 4 mm distal and slightly anterior to the femoral insertion of the PT. The graft was pulled through the fibular canal, and both ends were brought to the same length. The graft was secured within the fibular tunnel using a 6 × 20–mm PEEK interference screw. As in the LAR technique, the posterior graft limb was passed under the tendon of the biceps muscle and pulled through the femoral tunnels. A tensile force of 25 N was applied to each of the 2 graft limbs. Femoral fixation was achieved in each by inserting a 7 × 30–mm PEEK interference screw at 30° of flexion, without any external forces acting on the tibia. After previous aperture graft fixation, additional medial cortical fixations were carried out using a cortical button (Endobutton) (Figure 2) to guarantee position of the graft while testing.

To investigate the third hypothesis of the study, the test regimen was repeated with 2 additional femoral flexion angles for the fixation of the PFS strand in the ARC. While the LCL strand was fixed at 30° in all states, the PFS was alternatively fixed at 30°, 60°, and 90° of knee flexion, using the same tendon grafts.

### Statistical Analysis

Kinematic data were represented by degrees of angle for ER and VR in different states. Based on the limited number of specimens (n = 8), a nonnormal distribution was assumed. A Kruskal-Wallis test was conducted to examine whether there were significant differences between the groups. Wilcoxon signed rank tests were used to assess statistical significance between paired groups in different states. Statistical analysis was performed using IBM SPSS Statistics for Windows (Version 27.0). The significance level was set at *P* = .05.

## Results

**Figure 3.**
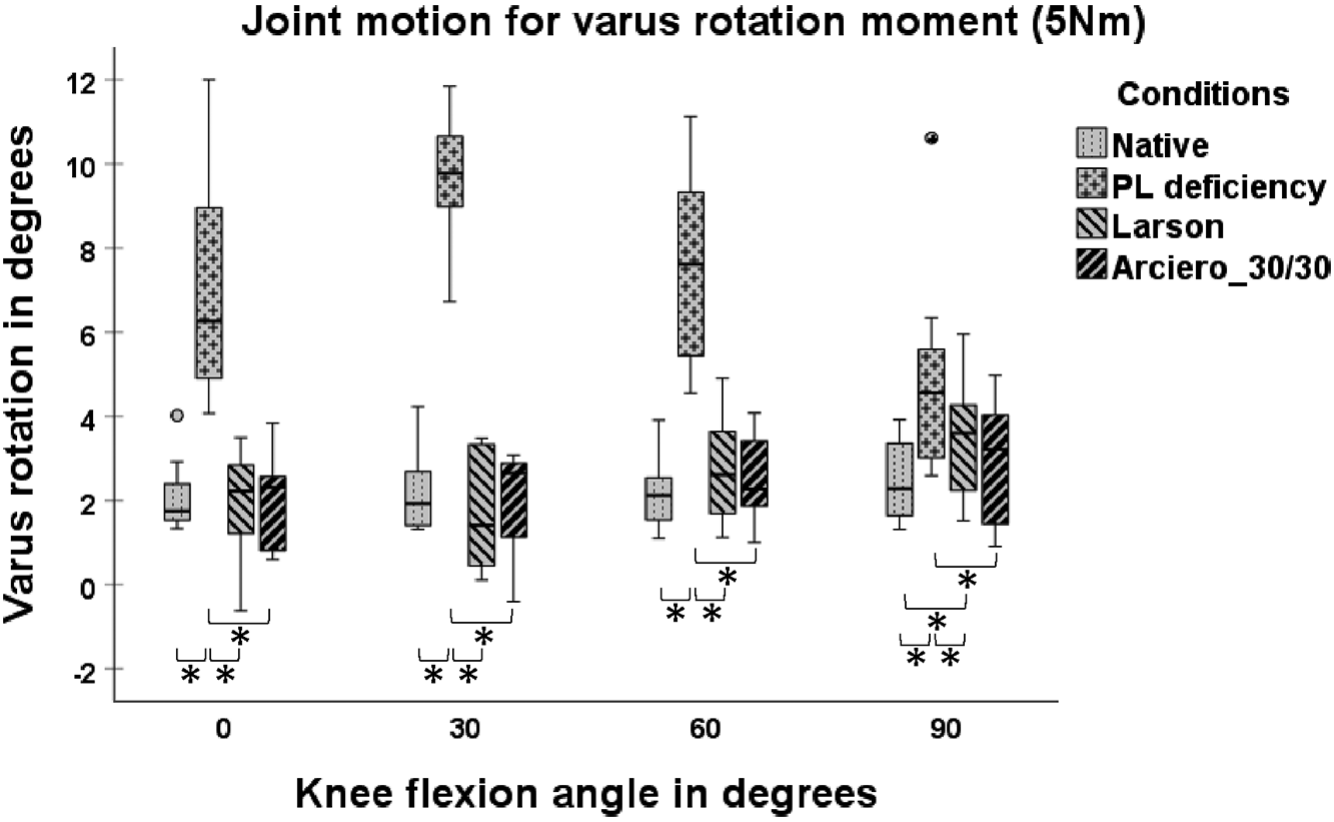
Joint motion with 5-N·m varus rotation torque in 4 states: native, posterolateral (PL) deficiency, Larson (LAR) and Arciero 30/30 (ARC30). Box plots show the median, interquartile range, and distributions (n = 8). Circles indicate outliers. Significant differences between states are marked with an asterisk (*) and a bracket indicating the 2 states.

At 90° of knee flexion, VR for the LAR was significantly higher in comparison with NAT (mean ± SD, 3.46°± 1.46° vs 2.47°± 1.01°; *P* = .012) (Figure 3).

**Figure 4.**
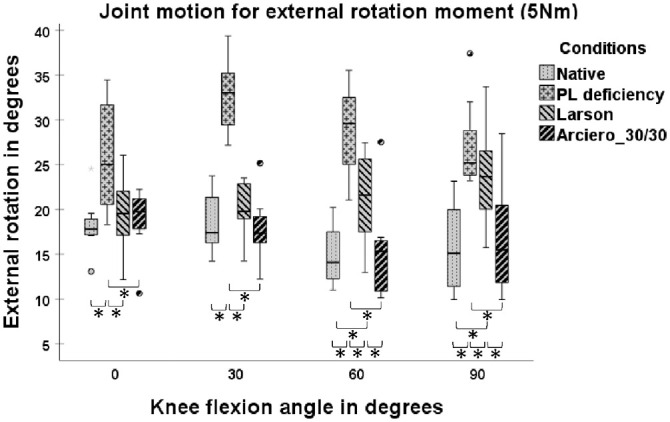
Joint motion with 5-N·m tibial external rotation torque in 4 states: native, posterolateral (PL) deficiency, Larson, and Arciero 30/30. Box plots show the median, interquartile range, and distributions (n = 8). Circles indicate outliers. Significant differences between states are marked with an asterisk (*) and a bracket indicating the 2 states.

The LAR showed significantly greater ER at 60° and 90° of knee flexion in comparison with NAT (21.24°± 5.14° vs 14.87°± 3.29° [*P* = 0.017]; 23.74°± 5.57° vs 15.77°± 5.07° [*P* = .012]) and in comparison with ARC30 (21.24°± 5.14° vs 15.39°± 5.58° [*P* = .017]; 23.74°± 5.57° vs 16.75°± 6.27° [*P* = .012]) (Figure 4).

**Figure 5.**
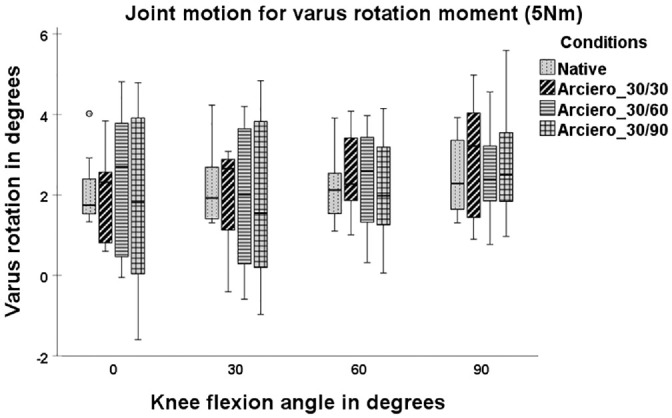
Joint motion with 5-N·m varus rotation torque in 4 states: native, Arciero 30/30, Arciero 30/60, and Arciero 30/90. Box plots show the median, interquartile range, and distributions (n = 8). Circles indicate outliers.

**Figure 6.**
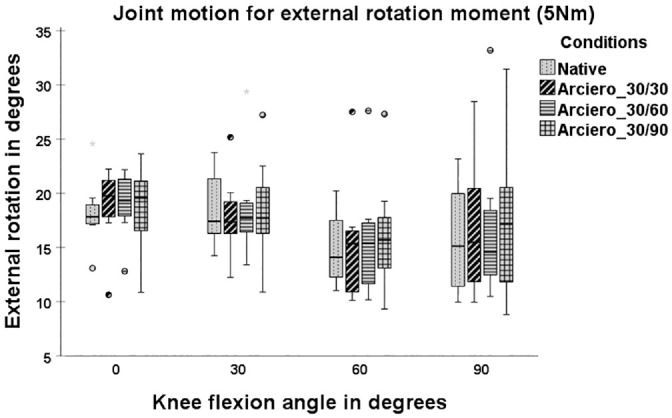
Joint motion with 5-N·m tibial external rotation torque in 4 states: native, Arciero 30/30 (ARC30), Arciero 30/60 (ARC60), and Arciero 30/90 (ARC90). Box plots show the median, interquartile range, and distributions (n = 8). Circles indicate outliers.

When the PFS fixation of the ARC was compared at 3 flexion angles (ARC30, ARC60, and ARC90), VR and ER did not significantly differ in comparison with NAT (Figures 5 and 6) at all flexion angles (*P*≥ .05). No significant differences in ER and VR were found among ARC30, ARC60, and ARC90 at any flexion angle (*P*≥ .05).

Kinematic data for all load cases and corresponding *P* values are listed in the Appendix (available in the online version of this article).

## Discussion

The main finding of this study was that the ARC technique restored ER and VR stability more closely to the NAT than the LAR technique at higher flexion angles. The first hypothesis was accepted, as the ARC was associated with greater tibial ER stability in comparison with the LAR. The second hypothesis was partially accepted, as both reconstruction techniques were able to restore VR native stability, except at 90° of flexion, at which the LAR was not able to restore VR stability to NAT. The third hypothesis was rejected, as all 3 flexion angles for PFS fixation of the ARC resulted in comparable ER stability.

In contrast to the LAR technique, the ARC represents a more anatomic reconstruction technique, with 2 femoral tunnels intended to restore the PT and LCL near to their native insertion points.^4,25^ When the LAR reconstruction is carried out, a nonanatomic single femoral tunnel is drilled at the most isometric point near the femoral epicondyle.^18^ It should be mentioned that determining the isometric point on the lateral femoral condyle can be challenging because a precise “isometric point” does not exist. The isometric point may vary owing to the complex biomechanics of the knee, which depend on the physiologic effects of rolling-gliding knee motion as well as the viscoelastic properties of ligaments, menisci, and the capsular cutaneous complex.^24^ A graphic analysis of cadaveric knees reported that even for the optimum isometric point, a mean displacement of 1.3 mm was found between specimens.^23^ This underlines the lack of reliability in determining the best femoral fixation spot when performing the LAR, and it may represent a weak point of this technique. Yet, the LAR technique may be more time-saving and less expensive, with only 1 femoral interference screw, in comparison with the ARC technique. From a biomechanical point of view, the ARC technique may allow better load sharing among the graft strands and may be better able to reproduce native external tibial rotation stability than the LAR technique. The LAR did not restore PLC stability at 60° and 90° of knee flexion. It is known from force measurement investigations that PT and PFL become the leading restraining structures against ER loads from 60° upward.^15,17,26^ These findings are reflected in the present results. When the ARC technique is used, the PFS acts as an autonomous structure, which may better reproduce PT and PFL function. Combined PLC and PCL reconstruction has been reported to have a high rate of residual posterolateral instability.^13^ For combined cruciate ligament lesions, recent literature recommends a single-stage combined reconstruction to enhance the synergism of these structures. As the ARC technique led to better external tibial rotation stability in comparison with the LAR, the ARC may better protect the combined cruciate reconstruction graft than the LAR.^7,12^

In the present study, the LAR was not able to restore VR stability at 90° of knee flexion. It is known that the primary restraint against VR torque from 0° to 90° of flexion is the LCL, while the PT and PFL play only minor roles.^17^ One possible explanation for this might be that the anterior tendon strand in the LAR follows the anatomic path less than the anterior strand in the ARC and may therefore be less tensioned at higher flexion angles. Another difference between the techniques is the fibular screw fixation performed with the ARC. Although outside the scope of the current analysis, it may be hypothesized that the fibular fixation allows less graft slippage during a flexion/extension cycle and is therefore more stable.

Fixation of the PFS at 30°, 60°, and 90° of knee flexion in the ARC technique did not show any significant differences in relation to ER or VR stability. These findings are in contrast with previous assumptions based on the highest mean load response for ER for the PT and the PFL (13 N for the PFL and 12 N for the PT) recorded at 60° of knee flexion.^17^ The graft should be fixed at the flexion point that shows the highest load response per applied torque.^17,21,28^ For this reason, some surgeons prefer fixation of PFS at 60° or 90° of flexion. Based on the present results, it must be assumed that performing femoral fixation of the PFS at different degrees of flexion does not have any influence on the posterolateral stability of the knee joint. However, the PFS may not completely follow the anatomic direction of the PT and may therefore be loaded differently from native measured PT loads.

It is still unclear whether fibular screw fixation is required with the ARC technique. As mentioned earlier, the fibular screw may provide better stability against VR torque, but this still needs to be investigated in further biomechanical studies. There are also potential disadvantages, such as an intraoperative risk of fibular head fracture and higher costs of the fibular screw.

This study has some limitations. First, the specimens used for the present study were from older donors, in comparison with the typically younger patient population with PLC knee injuries. Second, this is a biomechanical in vitro study that reports on time-zero conditions, without taking into account graft-healing processes or postoperative graft elongation. Third, the grafts used in this study were bovine tendons rather than human grafts. However, bovine tendons have been shown to be a valid alternative to human hamstring grafts for experimental studies.^9^ Furthermore, specimens were not left/right pair matched. Yet, the order of the reconstruction types was alternated. Another limitation is that void filling with PMMA cement could have affected the results, as it may change material properties. Even the placement of a tibiofibular screw, which is necessary to ensure joint stability through the entire test protocol, is not physiologic and could have affected results. Moreover, multiple placements of the fibular screw in the third study objective with a nonrandomized order of the graft fixation angles could have affected the results. The posterolateral deficiency model may create a worst-case scenario, which may result in greater instability in comparison with clinical findings. Nonetheless, the posterolateral deficiency was uniform for all the specimens and was comparable with the ER and VR values in previous studies.^20^ It has to be mentioned that after statistical analyses with Wilcoxon signed rank tests, correction for multiple testing was not applied because our primary comparison was about the ARC versus the LAR and further comparisons (reconstructions to native or PLC deficiency and knee flexion angles for fixation of the ARC reconstruction) were regarded as secondary endpoints with a more explorative character.

This study biomechanically compares 2 commonly used fibular-based techniques for PLC reconstruction. In comparison to fibular-based techniques, tibiofibular-based PLC reconstructions have been reported to provide higher ER stability for higher degrees of flexion,^10,19,29^ while higher VR stability in 90° of flexion was reported for the LAR as compared with a tibiofibular-based technique.^20^ A recent review^5^ concluded that clinical outcomes and ability of restoring VR and ER stability were comparable between tibiofibular- and fibular-based reconstructions. This underlines that outcomes are inconclusive and further studies are required. Our results show that the less anatomic LAR technique was not sufficient to restore VR and ER stability at higher knee flexion, whereas the more anatomic ARC technique was able to restore the native state at all flexion angles. This might be interesting as fibular-based techniques are sometimes considered less demanding and more cost- and time-saving than tibiofibular-based reconstructions.

## Conclusion

The ARC technique was able to restore knee stability, with respect to ER and VR, more closely to the native uninjured state than the LAR technique at higher flexion angles. In addition, different femoral flexion angles for fixation of the PFS in the ARC procedure did not show any influence on posterolateral stability.

## Supplemental Material

sj-pdf-1-ajs-10.1177_03635465241294072 – Supplemental material for Posterolateral Knee Ligament Reconstruction Using the Arciero Technique Provides Greater Rotational Stability Than the Modified Larson Technique: A Biomechanical Study Supplemental material, sj-pdf-1-ajs-10.1177_03635465241294072 for Posterolateral Knee Ligament Reconstruction Using the Arciero Technique Provides Greater Rotational Stability Than the Modified Larson Technique: A Biomechanical Study by Christian Coppola, Maximilian Sigloch, Romed Hoermann, Michael Schlumberger, Philipp Schuster, Werner Schmoelz and Raul Mayr in The American Journal of Sports Medicine
