# Supplementary material for: Posterolateral Knee Ligament Reconstruction Using the Arciero Technique Provides Greater Rotational Stability Than the Modified Larson Technique: A Biomechanical Study
Source: Am J Sports Med. 2025 Jan 1;53(1):147–53. doi: 10.1177/03635465241294072 (PMC11689966; doi:10.1177/03635465241294072)
Supplement: sj-pdf-1-ajs-10.1177_03635465241294072 – Supplemental material for Posterolateral Knee Ligament Reconstruction Using the Arciero Technique Provides Greater Rotational Stability Than the Modified Larson Technique: A Biomechanical Study [file sj-pdf-1-ajs-10.1177_03635465241294072.pdf]

	Native 0° (2.09°±0.92)<br>Native 30° (2.2°±1.02)<br>Native 60° (2.18°±0.88)<br>Native 90° (2.47°±1.01)	PL deficiency 0° (7.04°±2.74)<br>PL deficiency 30° (9.68°±1.56)<br>PL deficiency 60° (7.56°±2.34)<br>PL deficiency 90° (4.94°±2.61)	Larson 0° (1.93°±1.32)<br>Larson 30° (1.75°±1.45)<br>Larson 60° (2.74°±1.31)<br>Larson 90° (3.46°±1.46)	Arciero30 0° (1.98°±1.14)<br>Arciero30 30° (2.00°±1.25)<br>Arciero30 60° (2.52°±1.05)<br>Arciero30 90° (2.91°±1.53)
**Native 0° (2.09°±0.92)**	-	0.012	0.889	1.000
**Native 30° (2.2°±1.02)**	-	0.012	0.624	1.000
**Native 60° (2.18°±0.88)**	-	0.012	0.208	0,575
**Native 90° (2.47°±1.01)**	-	0.012	0.012	0.327
**PL deficiency 0° (7.04°±2.74)**	-	-	0.012	0.012
**PL deficiency 30° (9.68°±1.56)**	-	-	0.012	0.012
**PL deficiency 60° (7.56°±2.34)**	-	-	0.012	0.012
**PL deficiency 90° (4.94°±2.61)**	-	-	0.012	0.050
**Larson 0° (1.93°±1.32)**	-	-	-	0.889
**Larson 30° (1.75°±1.45)**	-	-	-	0.889
**Larson 60° (2.74°±1.31)**	-	-	-	0,779
**Larson 90° (3.46°±1.46)**	-	-	-	0.327
**Arciero30 0° (1.98°±1.14)**	-	-	-	-
**Arciero30 30° (2.00°±1.25)**	-	-	-	-
**Arciero30 60° (2.52°±1.05)**	-	-	-	-
**Arciero30 90° (2.91°±1.53)**	-	-	-	-

Appendix table 1: Mean joint motion in degrees (°), standard deviation and and p-values with 5 Nm varus rotation torque comparing four states (Native, PL deficiency, Larson, Arciero 30°/30°).

	Native 0° (18.20°±3.19)<br>Native 30° (18.51°±3.26)<br>Native 60° (14.87°±3.29)<br>Native 90° (15.77°±5.07)	PL deficiency 0° (25.90°±6.08)<br>PL deficiency 30° (32.72°±4.05)<br>PL deficiency 60° (28.85°±4.98)<br>PL deficiency 90° (27.05°±5.01)	Larson 0° (19.46°±4.25)<br>Larson 30° (20.13°±3.04)<br>Larson 60° (21.24°±5.14)<br>Larson 90° (23.74°±5.57)	Arciero30 0° (18.81°±3.71)<br>Arciero30 30° (17.89°±3.71)<br>Arciero30 60° (15.39°±5.58)<br>Arciero30 90° (16.75°±6.27)
**Native 0° (18.20°±3.19)**	-	0.012	0.123	0.401
**Native 30° (18.51°±3.26)**	-	0.012	0.093	0.484
**Native 60° (14.87°±3.29)**	-	0.012	0.017	0.779
**Native 90° (15.77°±5.07)**	-	0.012	0.012	0.674
**PL deficiency 0° (25.90°±6.08)**	-	-	0.012	0.017
**PL deficiency 30° (32.72°±4.05)**	-	-	0.012	0.012
**PL deficiency 60° (28.85°±4.98)**	-	-	0.012	0.012
**PL deficiency 90° (27.05°±5.01)**	-	-	0.017	0.012
**Larson 0° (19.46°±4.25)**	-	-	-	0.484
**Larson 30° (20.13°±3.04)**	-	-	-	0.093
**Larson 60° (21.24°±5.14)**	-	-	-	0.017
**Larson 90° (23.74°±5.57)**	-	-	-	0.012
**Arciero30 0° (18.81°±3.71)**	-	-	-	-
**Arciero30 30° (17.89°±3.71)**	-	-	-	-
**Arciero30 60° (15.39°±5.58)**	-	-	-	-
**Arciero30 90° (16.75°±6.27)**	-	-	-	-

Appendix table 2: Mean joint motion in degrees (°), standard deviation and and p-values with 5 Nm external rotation torque comparing four states (Native, PL deficiency, Larson, Arciero 30°/30°).

	Native 0° (2.09°±0.92)<br>Native 30° (2.2°±1.02)<br>Native 60° (2.18°±0.88)<br>Native 90° (2.47°±1.01)	Arciero30 0° (1.98°±1.14)<br>Arciero30 30° (2.00°±1.25)<br>Arciero30 60° (2.52°±1.05)<br>Arciero30 90° (2.91°±1.53)	Arciero60 0° (2.33°±1.86)<br>Arciero60 30° (1.94°±1.87)<br>Arciero60 60° (2.37°±1.32)<br>Arciero60 90° (2.53°±1.20)	Arciero90 0° (1.84°±2.29)<br>Arciero90 30° (1.88°±2.14)<br>Arciero90 60° (2.13°±1.39)<br>Arciero90 90° (2.79°±1.45)
**Native 0° (2.09°±0.92)**	-	1.000	0.674	0.889
**Native 30° (2.2°±1.02)**	-	1.000	0.889	0.726
**Native 60° (2.18°±0.88)**	-	0.575	0.889	0.779
**Native 90° (2.47°±1.01)**	-	0.327	0.779	0.327
**Arciero30 0° (1.98°±1.14)**	-	-	0.484	0.575
**Arciero30 30° (2.00°±1.25)**	-	-	1.000	0.674
**Arciero30 60° (2.52°±1.05)**	-	-	0.575	0.441
**Arciero30 90° (2.91°±1.53)**	-	-	0.398	0.735
**Arciero60 0° (2.33°±1.86)**	-	-	-	0.208
**Arciero60 30° (1.94°±1.87)**	-	-	-	1.000
**Arciero60 60° (2.37°±1.32)**	-	-	-	0.327
**Arciero60 90° (2.53°±1.20)**	-	-	-	0.674
**Arciero90 0° (1.84°±2.29)**	-	-	-	-
**Arciero90 30° (1.88°±2.14)**	-	-	-	-
**Arciero90 60° (2.13°±1.39)**	-	-	-	-
**Arciero90 90° (2.79°±1.45)**	-	-	-	-

Appendix table 3: Mean joint motion in degrees (°), standard deviation and and p-values with 5 Nm varus rotation torque comparing four states (Native, Arciero 30°/30°, Arciero 30°/60°, Arciero 30°/90°).

	Native 0° (18.20°±3.19)<br>Native 30° (18.51°±3.26)<br>Native 60° (14.87°±3.29)<br>Native 90° (15.77°±5.07)	Arciero30 0° (18.81°±3.71)<br>Arciero30 30° (17.89°±3.71)<br>Arciero30 60° (15.39°±5.58)<br>Arciero30 90° (16.75°±6.27)	Arciero60 0° (19.01°±2.99)<br>Arciero60 30° (18.66°±4.72)<br>Arciero60 60° (15.80°±5.50)<br>Arciero60 90° (16.83°±7.22)	Arciero90 0° (18.63°±3.98)<br>Arciero90 30° (18.41°±4.81)<br>Arciero90 60° (16.22°±5.41)<br>Arciero90 90° (16.22°±5.41)
**Native 0° (18.20°±3.19)**	-	0.401	0.327	0.674
**Native 30° (18.51°±3.26)**	-	0.484	1.000	0.779
**Native 60° (14.87°±3.29)**	-	0.779	0.208	0.484
**Native 90° (15.77°±5.07)**	-	0.674	0.779	0.401
**Arciero30 0° (18.81°±3.71)**	-	-	0.674	0.575
**Arciero30 30° (17.89°±3.71)**	-	-	0.484	0.674
**Arciero30 60° (15.39°±5.58)**	-	-	0.674	0.401
**Arciero30 90° (16.75°±6.27)**	-	-	0.779	0.575
**Arciero60 0° (19.01°±2.99)**	-	-	-	0.441
**Arciero60 30° (18.66°±4.72)**	-	-	-	0.674
**Arciero60 60° (15.80°±5.50)**	-	-	-	0.484
**Arciero60 90° (16.83°±7.22)**	-	-	-	0.401
**Arciero90 0° (18.63°±3.98)**	-	-	-	-
**Arciero90 30° (18.41°±4.81)**	-	-	-	-
**Arciero90 60° (16.22°±5.41)**	-	-	-	-
**Arciero90 90° (16.22°±5.41)**	-	-	-	-

Appendix table 4: Mean joint motion in degrees (°), standard deviation and and p-values with 5 Nm external rotation torque comparing four states (Native, Arciero 30°/30°, Arciero 30°/60°, Arciero 30°/90°).
